# One Spatio-Temporal Sharpening Attention Mechanism for Light-Weight YOLO Models Based on Sharpening Spatial Attention

**DOI:** 10.3390/s21237949

**Published:** 2021-11-28

**Authors:** Mengfan Xue, Minghao Chen, Dongliang Peng, Yunfei Guo, Huajie Chen

**Affiliations:** 1 School of Automation, Hangzhou Dianzi University, Hangzhou 310018, China; xuemf@hdu.edu.cn (M.X.); 192060268@hdu.edu.cn (M.C.); gyf@hdu.edu.cn (Y.G.); chj247@hdu.edu.cn (H.C.); 2 HDU-ITMO Joint Institute, Hangzhou Dianzi University, Hangzhou 310018, China

**Keywords:** attention mechanism, object detection, YOLO, light-weight model, sharpening filter

## Abstract

Attention mechanisms have demonstrated great potential in improving the performance of deep convolutional neural networks (CNNs). However, many existing methods dedicate to developing channel or spatial attention modules for CNNs with lots of parameters, and complex attention modules inevitably affect the performance of CNNs. During our experiments of embedding Convolutional Block Attention Module (CBAM) in light-weight model YOLOv5s, CBAM does influence the speed and increase model complexity while reduce the average precision, but Squeeze-and-Excitation (SE) has a positive impact in the model as part of CBAM. To replace the spatial attention module in CBAM and offer a suitable scheme of channel and spatial attention modules, this paper proposes one Spatio-temporal Sharpening Attention Mechanism (SSAM), which sequentially infers intermediate maps along channel attention module and Sharpening Spatial Attention (SSA) module. By introducing sharpening filter in spatial attention module, we propose SSA module with low complexity. We try to find a scheme to combine our SSA module with SE module or Efficient Channel Attention (ECA) module and show best improvement in models such as YOLOv5s and YOLOv3-tiny. Therefore, we perform various replacement experiments and offer one best scheme that is to embed channel attention modules in backbone and neck of the model and integrate SSAM into YOLO head. We verify the positive effect of our SSAM on two general object detection datasets VOC2012 and MS COCO2017. One for obtaining a suitable scheme and the other for proving the versatility of our method in complex scenes. Experimental results on the two datasets show obvious promotion in terms of average precision and detection performance, which demonstrates the usefulness of our SSAM in light-weight YOLO models. Furthermore, visualization results also show the advantage of enhancing positioning ability with our SSAM.

## 1. Introduction

Convolutional neural networks have achieved great progress in the field of visual object detection and tracking by rich and expressive performance. Most researchers normally study its innovations in depth, width and structure [1,2,3]. In addition, the most important indicators for evaluating an object detector are accuracy and speed. As a guide, the visual object detector based on neural network can be divided into one-stage detector [4,5,6,7,8,9,10] and two-stage detector [11,12]. The most representative two-stage object detector is the R-CNN [13] series, which generally extracts the image feature by feature extraction networks, inputs the feature maps into region proposal network to generate regions of interest as first prediction and then makes classification and regression operations as second prediction. While the one-stage detector only passes through one prediction operation to perform the object detection task and combines classification and positioning together. Compared with the two-stage detector, the one-stage detector gains a substantial speed increase at the expense of a part of the accuracy. The most clearly direction for studying the one-stage detector is how to modify the network structure while maintaining its detection speed to enhance the detection accuracy and classification performance of the network.

Image filter is a traditional image processing method. It is an indispensable operation in image preprocessing. The quality of its processing effect will directly affect the effectiveness and reliability of subsequent image processing and analysis. In addition, the filters used for calculation are roughly divided into two categories: linear filters and nonlinear filters. Generally speaking, researchers will use image filter in processing datasets, but here we use it in the CNN structures, which is equivalent to assigning the weight of the training layer a fixed value. This is a good entry point. On this basis, we can do lots of follow-up research.

Research in recent years have shown that attention mechanisms such as SENet [14], ECANet [15] and CBAM [16] can bring positive effects to object detection, and its importance has also been widely studied. Attention mechanism leads the network to understand which information is important and which information is secondary, and distribute benefits to it. Our work is to improve the output effect of the light-weight YOLO models through attention mechanism. In this paper, we propose a new spatial attention module named “Sharping Spatial Attention” with image filter method and combine it with SE or ECA modules. We use this sharpening spatial attention to emphasize the extracted meaningful edge features. To this end, through various collocation and replacement experiments, we have obtained one suitable structure and method of the spatio-temporal attention in YOLOv5s model, so that intermediate feature maps can learn ‘which feature map is more useful in channel dimension’ and ‘where may exist objects in spatial dimension’, which aims to emphasize or suppress the visual information obtained by training.

First of all, by inserting our spatio-temporal sharpening attention module into YOLOv5s model, the model has shown an obvious improvement on the VOC2012 dataset. Based on this, we make various experiments to demonstrate its powerful. We try to find the best place to use the module and the appropriate structure of the module itself. To prove its widely applicability, we train and test YOLOv5s model with our SSAM on the MS COCO2017 dataset. The average precision shows good improvement and the speed is hardly affected. With this in mind, we speculate that the improve results come from the supplement of the edge features of medium and large objects and the emphasis on the edge features of small targets. So next we try to test the performance of our SSAM in YOLOv3-tiny model. The results are basically the same as using YOLOv5s model. But SE channel attention shows better performance than ECA channel attention in YOLOv3-tiny this time, maybe because of the influence of structure and complexity. All in all, experimental results on the two light-weight YOLO models prove the effective of our Spatio-temporal Sharpening Attention Mechanism. Moreover, the Sharpening Spatial Attention we proposed has a very small computational cost, while the ECA channel attention module has a small computational cost too, so in most cases the parametric and computational cost of our SSAM is negligible.

## 2. Related Work

In response to the problem of how to design a precise and fast detector, the YOLO series of visual target detectors turn out. The core idea of YOLOv1 [6] is to use the entire picture as the input of the network and return to the position of the bounding box and the category of the bounding box in the output layer directly. Although the detection speed of YOLOv1 is very fast, it is not as accurate as the R-CNN [13] detection method. YOLOv2 [7] proposes several improvement strategies to improve the positioning accuracy and recall rate of the YOLOv1 model while maintaining the detection speed: Batch Normalization [17], High Resolution Classifier, Convolutional with Anchor Boxes [11], Dimension Clusters, New Network: Darknet-19 [7], Multi-Scale Training and others. In addition, compared to YOLOv2, the main improvements of YOLO3 [4] include: adjust the network structure, use multi-scale features for object detection and replace softmax with Logistic for object classification. YOLOv3 draws on the residual network structure to form a deeper network level and multi-scale detection, which improves the detection effect of small objects and performs well on the MS COCO dataset. Then to YOLOv4 [18] and YOLOv5 in recent years, YOLOv4 has various degrees of optimization in terms of data processing, backbone network, network training, activation function, loss function, etc. It introduces core structures of PANet [19], SPPNet [20] and CSPNet [21] and divides the training method into bag of freebies, bag of specials and other optimization and improvement methods for deep CNNs. In addition, then Ultralytics comes up with YOLOv5. Although it does not substantially change the structure of network, its pytorch-based deep learning framework is easy for researchers to study, and its excellent convergence speed and accuracy in training made this structure a SOTA in May last year. In addition, our experiments on light-weight YOLO models are also based on this network.

Attention mechanism has proven to be a potential mean to enhance deep CNNs [15], which can be simply divided to three classes: channel attention, spatial attention and spatio-temporal attention with the former two. SENet [14] presents an effective mechanism for the first time to learn channel attention and shows great performance in various deep CNNs. CBAM [16] combines avgpool and maxpool in SE channel attention and puts forward spatial attention using avgpool and maxpool in another dimension. GSoP [22] counts the covariance matrix before performing pooling and acquire more rich features from input. GE [23] uses a depth-wise convolution to aggregate features and explores spatial extension. GCNet [24] thoroughly discusses the advantages and disadvantages of Non-local method and then introduces Non-local module with SE module to take advantage of the global context. ACNet [25] improves the model’s robustness to image flipping and rotation and obtains better feature expression. DANet [26] starts from the enhancement of global feature fusion and the correlation between semantic feature qualities, then puts forward the methods of position attention and channel attention mechanism. ECANet [15] aims at capturing local cross-channel interaction information, it investigates a 1D convolution with adaptive kernel size to replace FC layers in channel attention module. The experiments prove the ECA module achieves better performance than SE module in most cases and it is lightweight. In addition, for our method, we pay more attention to the edge information of the image and propose a simply but efficient spatial attention method fusing with channel attention. Compare with CBAM, our method shows better performance in light-weight YOLO models with low model complexity.

## 3. Proposed Method

By observing some detection results, we found that the reason for the misjudgment or low accuracy of object detectors is that the edge features in the extracted feature maps are not obvious, and the edge judgment is blurred when predicting the objects, which leads to a phenomenon that some parts of objects are not framed by anchor boxes. If we can use some methods to strengthen these edge features, the object location may be more accuracy, so it may be a suitable point to introduce sharpening filter by convolutional operation, which is the first motivation to construct the sharpening method. In the meanwhile, if we introduce some image filters to strengthen details of the intermediate feature maps, some noises and background structures will inevitably be strengthened. We try to add appropriate channel attention modules to reduce the impact. Under the influence of the CBAM attention mechanism, we notice that it is a good idea to combine channel attention and spatial attention and study their combinations. The spatial attention module in CBAM truly bring a positive effect to networks such as ResNet50 [27] and ResNeXt101 [28]. However, CBAM cause a negative impact in our experiments using light-weight model YOLOv5s, which can be seen in Table 1. Compared to SE attention module, CBAM adds a maxpool branch in channel attention and constructs one new spatial attention module using an adaptive a convolution operation. We infer that the spatial attention module in CBAM may be not suitable for YOLOv5s. Therefore, we try to design a spatial attention that can have a positive impact and we think it is a good try to add edge operation in form of spatial attention, which is another motivation, and we try to verify the usefulness of sharpening spatial attention.

Basically speaking, with some intermediate feature maps from different structures, the best scheme of SSAM can be divided into two parts: channel part and spatio-temporal part. Since sometimes sharpening spatial module may not work well in some structures. For major blocks in backbone and neck, we use the channel part. For output heads, we use the spatio-temporal part. For instance, if the network acquires one input intermediate feature map I ∈ R^C×H×W^, where C, H and W represent channel, width and height. Attention of the channel part can be learned by:

(1) $$\begin{array}{c} I_{c}=M_{c}\left(I\right)\;⊗\;I \end{array}$$

Attention of the spatio-temporal part can be learned by:

(2) $$\begin{array}{c} I_{\mathrm{sc}}=M_{s}\left(M_{c}\left(I\right)\right)\;⊗\;I \end{array}$$

where M_c_ and M_s_ are channel attention method and spatial attention method, respectively. ⊗ denotes element-wise multiplication. I_c_ and I_sc_ are the refined outputs of two parts. The detailed information of each attention method is described below.

### 3.1. Spatial Attention Module

**Figure 1:**
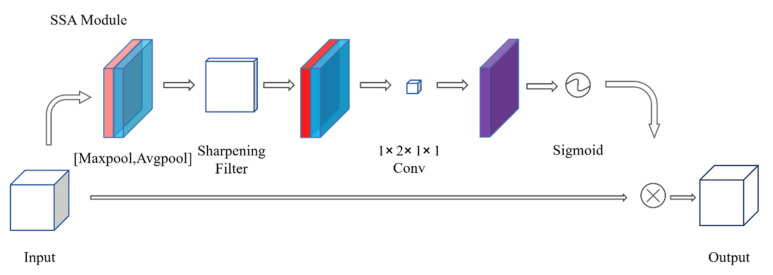
Our proposed SSA module.

We propose a spatial attention mechanism which incorporates sharpening filters: Sharpening Spatial Attention (SSA). Different from spatial attention module in CBAM, SSA module focus on ‘where’ the edges of the object should be and ‘how much’ to strengthen the edges for locating better, which is a supplementary enhancement for targets and can be affected by channel attention. The structure of our SSA module is showed in Figure 1.

We use maxpool and avgpool to compress spatial dimension information of the input feature maps and concentrate them, then let it go through a sharpening filter to enhance the edge feature information. Next, set up a layer of convolution to reduce the channel of feature maps and pass through a sigmoid activation function. Finally, multiply the spatial weights to the original input feature maps. To sum up, our Sharpening Spatial Attention module is computed as:

(3) $$\begin{array}{c} M_{s}\left(I\right)\;=\sigma \left(W_{1\times 1}\left(f^{n\times n}\left(\left[\mathrm{Avgpool}\left(I\right);\mathrm{Maxpool}\left(I\right)\right]\right)\right)\right)\\ =\sigma \left(W_{1\times 1}\left(f^{n\times n}\left(\left[I_{\mathrm{avg}}^{s};I_{\max}^{s}\right]\right)\right)\right) \end{array}$$

where σ denotes the sigmoid activation function, f^n×n^ represents the sharpening convolution operation with the filter size of n × n and W_1×1_ is a 1 × 2 × 1 × 1 parametric convolution, whose channel is equal to the channel of feature maps.

In order to emphasize the edge information of the extracted features, we introduce image sharpening filter. The main purpose of the image sharpening is to highlight the transition part of the grayscale and enhance the details in the image. Here we use it in deep CNNs and test three different operators: 3 × 3 s-order Laplace sharpening, 5 × 5 s-order Laplace sharpening and 3 × 3 first-order Sobel sharpening. Let the output of pooling operation goes through a sharpening filter and give it a weight while reduce the channel of feature maps, so the network can adjustment the information of the object edges.

### 3.2. Channel Attention Module

Through global information, the channel attention module leads the network to selectively enhance features containing useful information and suppress useless features. It is known that SE module is a classic channel attention module. Hu et al. [14] first perform an avgpool operation on the feature map obtained by convolution to collect the channel-level global feature. Then perform an excitation operation called MLP on the global features to learn the relationship between each channel and also obtain the weights of the different channels. Next, come through a sigmoid activation function. In addition, finally score the channels of the original feature map. SE module is effective and adaptive, but sometimes it may cause the accumulation of parameters and waste of computing power.

**Figure 2:**
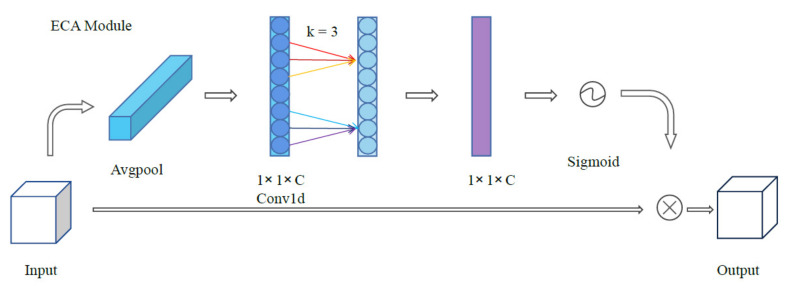
The ECA module [15].

Wang et al. [15] put forward ECA module as an innovation of SE module, which improves the information exchange between channels while reducing the amounts of parameters. They believe that dimensionality reduction and cross-channel interaction have brought side effects to channel information, and it is proved by several experiments that avoiding dimensionality reduction helps to learn effective channel attention. As shown in Figure 2, the idea of ECA module is very creative, which removes the MLP layer in SE module and learns channel information directly through a 1D convolution after global average pooling. In short, SE module and ECA module are computed as:

(4) $$\begin{array}{c} M_{c}^{\mathrm{se}}\left(I\right)\;=\sigma \left(\mathrm{MLP}\left(\mathrm{Avgpool}\left(I\right)\right)\right)\\ =\sigma \left(W_{r}\left(W_{d}\left(I_{\mathrm{avg}}^{c}\right)\right)\right) \end{array}$$

(5) $$\begin{array}{c} M_{c}^{\mathrm{eca}}\left(I\right)\;=\sigma \left(C1D_{k}\left(I_{\mathrm{avg}}^{c}\right)\right) \end{array}$$

where σ denotes the sigmoid activation function, MLP represents multi-layer perceptron with one hidden layer, W_r_ and W_d_ are the MLP weights and C1D indicates a 1D convolution. ECA generates channel weights by performing a fast 1D convolution of size k, where k is determined via a mapping of channel dimension C [15]. Note that here we set the kernel size of C1D to 3.

## 4. Experiments

### 4.1. Object Detection Test on VOC2012 Dataset

In this subsection, we explore the appropriate structure and location of our SSAM on object detection task using VOC2012 dataset. We use YOLOv5s as the basic model and one RTX2080ti as GPU. The number of images for training and validation are 5717 and 5823. We firstly test different locations of three attention mechanisms in YOLOv5s model and mainly compare the combination effect of SENet, ECANet and CBAM with our SSA module. Then try to change the details of our SSA module to find an appropriate plan. Note that our training uses most of the rules and options as same as training on MS COCO2017 dataset to acquire more precise results in the next step. The size of the input images in the experimental part are all 640 × 640, and the weights are trained in condition of confidence threshold: 0.001 and NMS threshold: 0.6. This may not the best choice of two thresholds, but here we need to unify the train conditions of these data, so we just choose a suitable scheme. One more thing, here we use HardSwish (in Bottleneck) and LeakyReLU (in BottleneckCSP) as basic activation functions in YOLOv5s model.

#### 4.1.1. Comparison Using Different Fusion Methods and Different Attention Mechanisms

**Figure 3:**
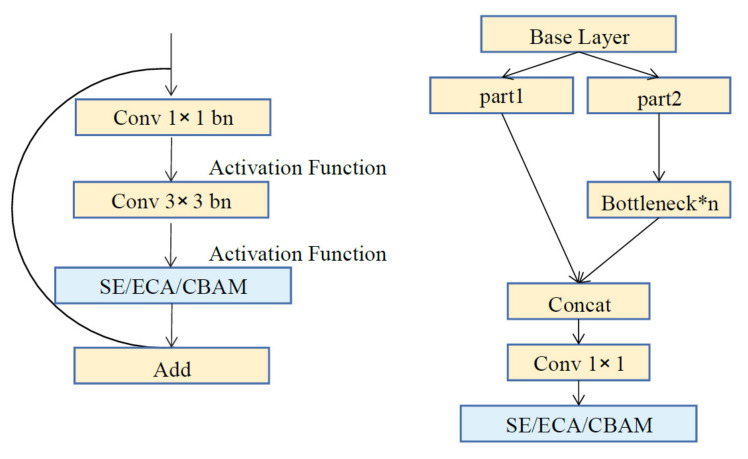
Two different fusion methods.

First of all, we test the performance of SENet, ECANet and CBAM attention mechanisms in YOLOv5s model using two different fusion methods as presented in Figure 3. The left one is an application in small Bottleneck block, and the right one is a combination with BottleneckCSP block. In our view, introducing too many attention modules into a light-weight YOLO model may be counterproductive.

The experiments show the advantage of the latter one in our condition. Using YOLOv5s model as the basic detector, the experimental results are shown in Table 1. Either SE module or ECA module can improve the performance of object detection by a clear margin with method on the right side. Meanwhile, ECA modules outperforms SE modules by 0.6% and 0.1% in terms of AP50 and AP50:AP95, respectively. Another discovery, CBAM reduces the average precision of the network. Maybe the spatial attention module in CBAM is not suitable for this situation. We infer that with too more attention modules in the backbone the model may lose its ascendancy in speed and accuracy, or method on the left side just add useless complexity. Note that epoch times on VOC2012 dataset are 400 with batch size 20 and these data are from the best weight in training.

#### 4.1.2. Comparison Using Different Structures of Spatio-Temporal Sharpening Attention Mechanism

From what we discussed above, we use the fusing method on the right side to locate attention mechanisms in YOLOv5s model and now we try to find how to combine our SSA module with channel attention mechanisms. For research, here we divide all BottleneckCSP in YOLOv5s model into three parts: backbone part (3 blocks), neck part (2 blocks) and head part (3 blocks). We can change the locations of our SSA module and channel attention module to test the performance of our method, and we can test where to set our spatio-temporal module. To deal with, we set several different methods to introduce channel attention module and spatial attention module into three different parts. Note that option parameters here are the same as in former experiment to unify the conditions and here we use Laplace 5 × 5 as the sharpening filter in our SAA module.

As presented in Table 2, by combining with ECA modules, SSA module has achieved great promotion on object detection. It is learned from experiments that our SSA module cannot be used more than once in the backbone. If we put SSA modules into the backbone neural network, the training process cannot complete the convergence smoothly. We infer that adding the edge enhancement modules to the feature extraction network will cause the weight of the edge part to fluctuate up and down, which may cause convergence difficulties in training. In addition, the neck part is also not a suitable place to place our SSA module. The best scheme in Table 2 is to place our SSAM module in the head part and implant ECA modules in other parts. We also test the performance of SAM module (the spatial module of CBAM) in the head part, but it cannot work well. Under the settings of best combination with ECA modules, YOLOv5s model with our SSA module is superior to the original YOLOv5s model 2.4% and 1.6% in terms of AP50 and AP50:AP95, respectively. Meanwhile, the best combination of ECA and SSA modules achieves 1.2% and 1.1% gains over only use ECA modules in terms of AP50 and AP50:AP95, respectively. On the other side, it is better to let the input feature map pass through the two attention modules in the order of channel attention and spatial attention, but actually there is not much difference between the two orders.

**Figure 4:**
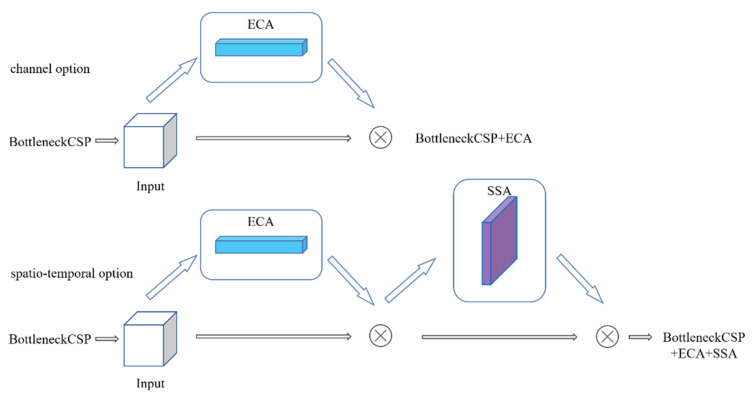
Two different parts of attention.

As mentioned before, the optimum application method of attention mechanism we find on VOC2012 dataset can be summarized into two parts as shown in Figure 4: channel part and spatio-temporal part. Note that maybe other channel attention modules will do better than ECA module, but here we just test ECA and SE modules.

In addition, from Table 2, we find that if we don’t put ECA modules in backbone or neck, SSAM module may not work as expected. The best scheme outperforms the one without ECA modules in backbone and neck by 1.6% and 1.4% in terms of AP50 and AP50:AP95, respectively. In addition, if we just use SSA in head, the scheme with ECA modules in backbone or neck is also better. We predict that ECA modules in backbone or neck can reduce the impact of unnecessary noise in the feature maps, so our SSA module can work well. Since if there exist some clear noises, SSA module may enhance noises while enhancing edge features. This is a very important discovery and prediction, so if we want our SSA module performs better, we need to add some channel attention modules in the former structures to reduce the influence of noises or backgrounds. Furthermore, we test the scheme using No Sharpening Attention (NSA) which means SSA without sharpening filter as an ablation experiment, and the NSA module performs similar to SAM and has no positive effect, which verify the importance of sharpening filter in SSA. The experiments also prove that the combination of our proposed SSA module and channel attention modules can form a good spatio-temporal attention mechanism.

#### 4.1.3. Comparison Using Different Edge Operators

After determining the location of our SSA module and its combination with the channel attention modules, we need to conduct an experimental analysis on the internal structure of our SSA module to determine the appropriate edge operator. Edge detection is used in the process of image sharpening in our SSA module, which is a research field in image processing and computer vision. The essence of sharpening filter is to use the edge detection operator to strengthen the edges of the original image, and the essence of edge detection algorithm is to find areas with relatively large grayscale changes, but the edges of the object are not always ideal edges, they are usually affected by some shadows, partial specular reflection or diffuse reflection near the edges of the object. So different edge operators cause different results.

Here we test two classic edge detection operators: Laplace operator and Sobel operator. The Sobel operator usually contains two sets of 3 × 3 matrices, which are divided into horizontal and vertical. After convolving them with the image, the approximate value of the horizontal and vertical brightness difference can be obtained. The Sobel operator has a smoothing effect on noise and provides more accurate edge direction information, but the edge positioning accuracy may be not high enough. Different from the first-order Sobel operator, the Laplace operator is a second-order edge detection operator usually compose of a set of 3 × 3 or 5 × 5 matrix. Compared with the Sobel operator, the Laplace operator has a better edge recognition effect, but the Laplace is sensitive to noise and may have negative effects, so the performance may be better after combining channel attention mechanisms. The previous experiments just randomly select one 5 × 5 template of Laplace operator for testing to acquire more refined edges. From Table 3, all the three edge detection templates can significantly improve the final detection accuracy, and the 5 × 5 template of Laplace operator we select above has the best detection effect by coincidence. However, further test and research are needed at this step, our scheme is simply one of the suitable plans.

#### 4.1.4. Comparison Using Different Methods of Extraction

For the perspective of multi-channel spatial information extraction, Woo et al. [16] test the extraction effects of maxpool and avgpool in CBAM. Among them, maxpool is to extract the spatial pixels points with the largest value in the full channel into the output spatial dimension, and avgpool is to calculate the average spatial pixels of the full channel and then put it into the output spatial dimension. In our view, both extraction methods have their advantages and disadvantages. Maxpool can improve the presence of some small and medium targets in the output maps. However, it may also introduce some unnecessary and irrelevant pixel groups. So simply using maxpool may not be good to improve the detection accuracy. Avgpool highlights the points with higher average pixel values in all spatial dimensions. This extraction method can filter some noises and backgrounds, but relatively speaking, sometimes it may weaken some spatial information we need. As shown in Table 4, we test the detection performance of our SSA module using maxpool, avgpool and max&avgpool on the premise that the edge operation and combination method have been determined. The results of experiments are consistent with our inferred theory above. Using maxpool alone can acquire 0.4% and 0.3% gains over using avgpool alone in terms of AP50 and AP50:AP95, respectively. However, the performance of combining two extraction methods in our SSA module is obviously better than using one extraction method alone.

### 4.2. Object Detection on MS COCO2017 Dataset

After testing the structure and performance of our method on the VOC2012 dataset, we need to verify the effectiveness of our Spatio-temporal Sharpening Attention Mechanism on the MS COCO2017 dataset. Compared with the VOC2012 dataset, the MS COCO2017 dataset has added a lot of objects of small and medium size, which are difficult to detect. In addition, in various images, they will be mixed with some large objects. This is also the characteristic of MS COCO2017 dataset, which is frequently used to determine the performance of object detectors today. There are 118,287 training images and 5000 validation images in the MS COCO2017 dataset. We evaluate the experimental performance of our SSAM in YOLOv5s and YOLOv3-tiny models on the MS COCO2017 dataset. Here we use one RTX3090 GPU. For YOLOv5s model, we set batch size as 48 and train for 400 generations. The default threshold in the NMS non-maximum processing method is set to 0.65, but we also test other thresholds, and then write the best result in the table. Note that all activation functions in YOLOv5s model we use here are SiLU activation function. For YOLOv3-tiny model, we set batch size as 64 and train for 300 generations. The size of the input images in the experimental part are all 640 × 640. Other options remain unchanged. [Project URL: https://github.com/cmh707122660/SSA-SSAM]

#### 4.2.1. Model Changes for YOLOv3-Tiny

For the combination method of YOLOv5s model with our SSAM, we have evaluated and adjusted it on the VOC2012 dataset. Here we just use MS COCO2017 as dataset to further verify the performance of our proposed algorithm. For YOLOv3-tiny model, we adopt a similar combination method to YOLOv5s model. Since YOLOv3-tiny does not have the insertion space in the neck part, we first add ECA channel attention module after channel upgrading in the backbone, and then add channel attention module and our SSA module in the output heads in turn. Note again that the Spatio-temporal Sharpening Attention Mechanism here is not necessarily the best processing scheme, but we can verify the effectiveness of our method through these results.

#### 4.2.2. Comparison of mAP, Speed and Weight

From Table 5 and Table 6, we know that our Spatio-temporal Sharpening Attention Mechanism performs very well on the MS COCO2017 dataset. For YOLOv5s model, the combination of ECA and SSA modules have an excellent improvement performance in terms of average precision, which reaching 2%, 1.9% and 0.9% on AP50, AP75 and AP50:95, respectively. The detection speed of YOLOv5s with our SSAM can reach 435FPS. Moreover, after fusing our method, the object detector’s detection speed, computational complexity, model parameters, detection weights and other relative parameters do not fluctuate too much, which means that our SSA module conforms to the requirement of speed and accuracy and can be implemented in actual projects. From the point of channel attention, SE channel attention performs significantly inferior to ECA channel attention in YOLOv5s model. It needs more computing power and parameters but does not acquire better detection speed and accuracy.

For YOLOv3-tiny model, the combination of SE and SSA modules achieve 1.1%, 1% and 0.7% improvement in terms of AP50, AP75 and AP50:95, respectively. The combination performance of using SE channel attention modules is slightly better than using ECA channel attention modules in terms of average precision. According to our speculation, the difference in image feature extraction of YOLOv3-tiny model and YOLOv5s model may have affected our spatio-temporal method. However, the disadvantages of SE channel attention in terms of speed and parameter still exist. In brief, the results of experiments in MS COCO2017 dataset also prove the efficient of our Spatio-temporal Sharpening Attention Mechanism in light-weight YOLO models.

#### 4.2.3. Detection Results of YOLOv5s with SSAM on COCO2017 Dataset

**Figure 5:**
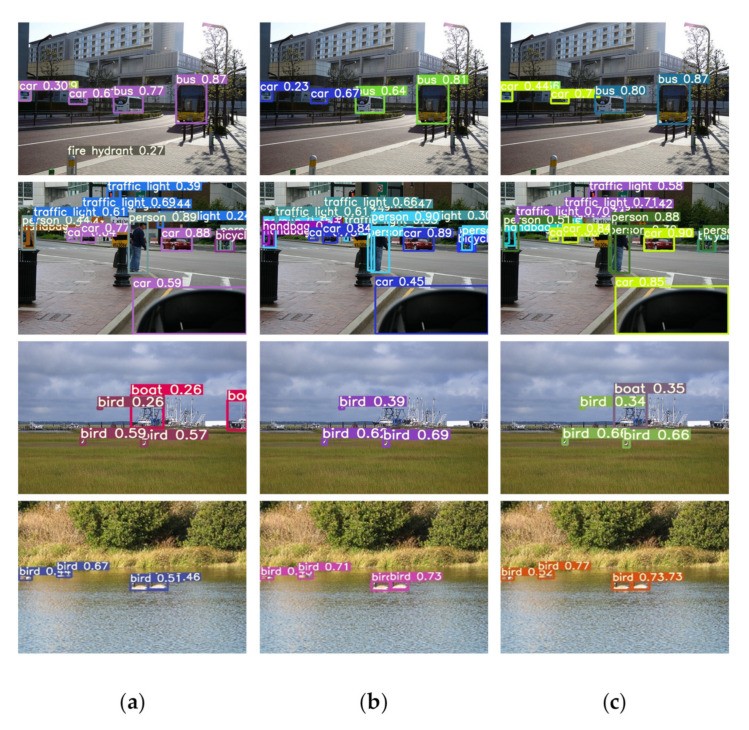
Detection results of YOLOv5s model with SSAM: (**a**) YOLOv5s; (**b**) YOLOv5s+ECA; (**c**) YOLOv5s+ECA+SSA.

In the MS COCO2017 dataset, object sizes are roughly divided into three types: small, medium and large. In order to explore and verify the effectiveness of SSA spatial sharpening attention and SSAM spatio-temporal sharpening attention mechanism. Here, the detection results of different size are compared in Table 7 and Table 8, which show the average precision of three sizes of objects obtained in the experiments. It is inferred that our deduction of the SSA module at the theory part is relatively correct, embedding the SSA module can improve the edge information of large objects, which can be seen in the visualization results. In addition, from Table 7 and Table 8, our SSA module can also improve the presence of small and medium-sized objects, thus promote the increase of AP_small_ and AP_medium_. As shown in Table 7, the average precision of the YOLOv5s+ECA model with SSA for small, medium and large objects has increased by 2.6%, 0.8% and 0.7%, respectively. In addition, the average precision of small objects is mainly improved in the experiments with our SSA module. Judging from the actual detection images, as shown in Figure 5, our SSAM can significantly improve the confidence of detecting objects of various sizes, especially objects with clearer edge contours.

#### 4.2.4. Visualization of YOLOv5s with SSAM on COCO2017 Dataset

**Figure 6:**
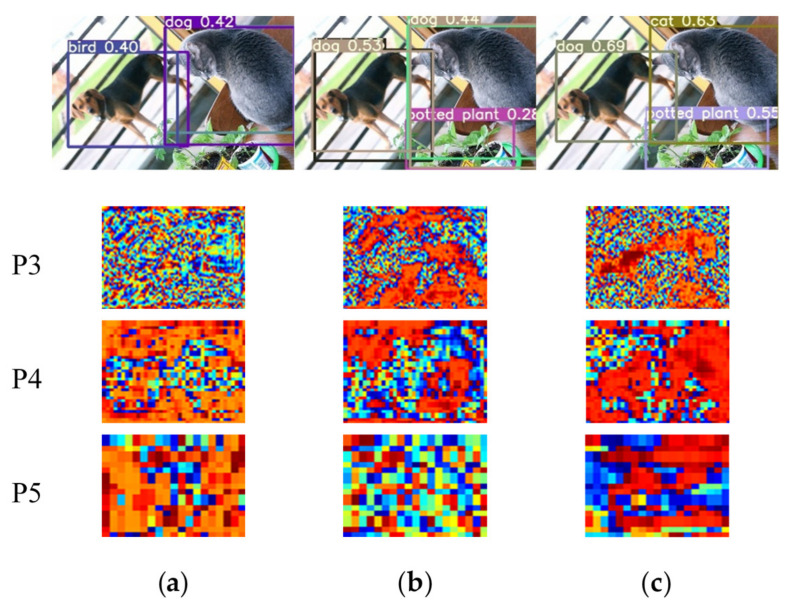
Visualization 1 of YOLOv5s model with SSAM: (**a**) YOLOv5s; (**b**) YOLOv5s+ECA; (**c**) YOLOv5s+ECA+SSA.

**Figure 7:**
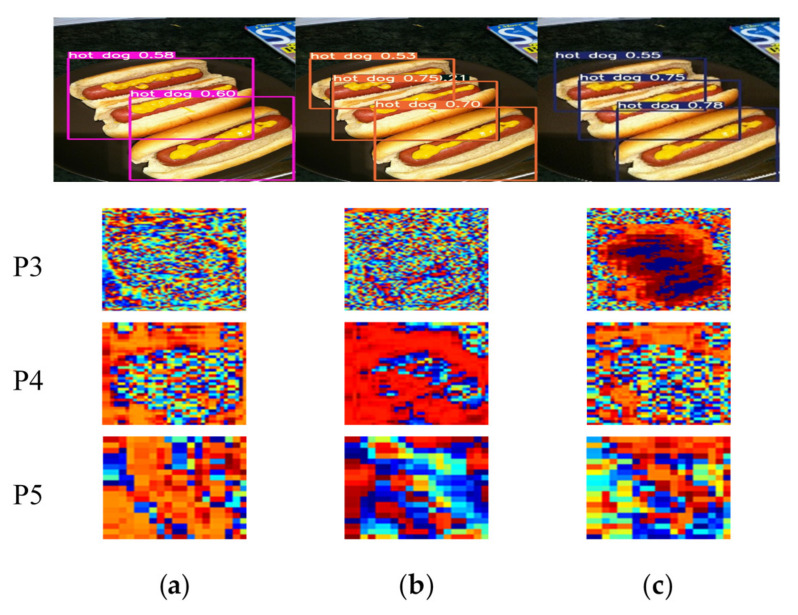
Visualization 2 of YOLOv5s model with SSAM: (**a**) YOLOv5s; (**b**) YOLOv5s+ECA; (**c**) YOLOv5s+ECA+SSA.

**Figure 8:**
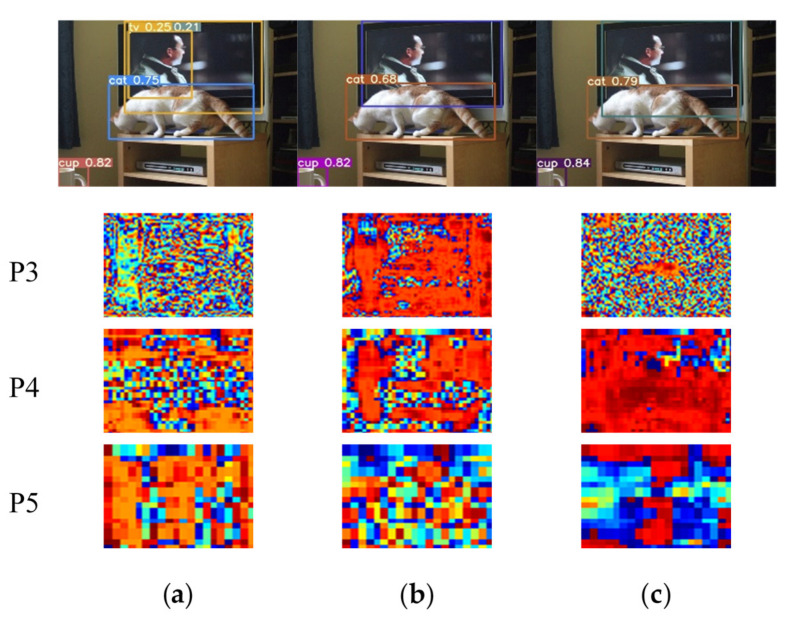
Visualization 3 of YOLOv5s model with SSAM: (**a**) YOLOv5s; (**b**) YOLOv5s+ECA; (**c**) YOLOv5s+ECA+SSA.

In this subsection, we need to verify the innovation and starting point of our SSA module through reliable visualization results. Since the phenomenon of object edge loss mentioned in the theory part is an empirical assumption after we have observed many detection images and results. It is mainly observed that lots of objects, especially the edges of small and medium-sized objects, jump out of the prediction boxes, which is one of the important reasons for the low IOU. We set the visualization node on the three output heads of YOLOv5s, that is, behind the BottleneckCSP blocks embedding with the spatio-temporal sharpening attention mechanism SSAM. According to the design of the neural network, the sizes of three output feature maps are 80 × 80, 40 × 40 and 20 × 20 corresponding to 640 × 640 input images. Here they are resized to a same size in order to facilitate unified observation. The visualization results can be seen in Figure 6, Figure 7 and Figure 8.

It is known that the three output YOLO heads correspond to different receptive fields and detection sizes. The shallower output head P3 has a larger feature map, and its ability to locate objects is more powerful, which is generally used to detect small objects. P4 can be used as a transition to detect objects of medium size. P5 is the deepest YOLO head, which is mainly used to identify large objects that take up more space in the image. As shown in Figure 6, our SSA module can make the network pay more attention to the position of the object themselves in spatial dimension, especially on the shallow output head P3. The addition of our SSAM mechanism makes the network focus on the dog, cat and plant, and basically draws complete outlines of dog and cat with obvious edge contours. Compared to YOLOv5s model with ECA modules, our SSAM can also lead the feature maps to reduce the weight on the background structure. The accurate positioning information in the P3 head means that the model’s ability to locate objects is better, which is basically the same as our inference of the SSA module during the experiments.

By observing the visualization results of Figure 7 and Figure 8, we have found an interesting phenomenon. The main purpose of our SSAM spatio-temporal module is to enhance the edge information of the extracted object features to improve the average precision of object detection. However, after adding our SSAM mechanism, in the P3 head, the spatial information of the entire object is enhanced, thus showing a more complete object contour in the P3 visualization. Our speculation is that when the network perceives that the strengthening of this part of the edge is conducive to target positioning, the inner edge will continue to radiate to the center, thereby obtaining more complete object information in spatial dimension. From this point of view, our SSA module also has the ability to find the spatial position of objects.

#### 4.2.5. How to Plug into Other Light-Weight Detectors

The sections above are the detection results and visualization results in the light-weight YOLO networks, which basically verifies that our hypothesis is valid and our method is effective. We also conclude the embedding position and application method of our sharpening spatial attention SSA in the neural network. Here it is necessary to discuss how it can be applied to other light-weight networks as a baseline. Our spatio-temporal sharpening mechanism SSAM is not exclusive to the YOLO models. Our SSA module uses a fixed sharpening filter template, and the characteristics of sharpening the edges of objects and focusing on the spatial information of the objects do not change with the change of the model. Therefore, combining with our detection results and visualization results on the VOC2012 and COCO2017 datasets, we believe that the spatio-temporal part of our spatio-temporal part of SSAM should be plugged before the down sampling structure and output feature maps of other light-weight networks, especially at the deep layer. In the experiments, we found that our SSA module at deep layer can promote the enhancement of objects better. The embedding of the channel part varies from network to network. The benchmark here is to insert the channel part after each main block in other backbone networks. The specific application in other networks needs further research.

## 5. Conclusions

In this paper, we set an empirical assumption about the loss of edge information of the object and focus on strengthening spatial edge information for deep CNNs with low computational complexity and parameters. To this end, we propose Sharpening Spatial Attention (SSA) module, which generates spatial attention through an image filter, whose value of weight is determined by one edge detection operator. Furthermore, we propose one Spatio-temporal Sharpening Attention Mechanism (SSAM) to combine our SSA module with channel attention modules in order to perform better. Experimental results demonstrate that our SSAM is a lightweight and efficient method to improve the performance of YOLO architectures such as YOLOv3-tiny and YOLOv5s. In the visualization results, we verify the inference that strengthening the edge information by the way of spatial attention mechanism helps to strengthen the network’s ability to locate objects of various sizes in spatial dimension. On the one side, our method can improve the average detection precision with low loss, which is of great significance for the application of object detection in some fast scenes. On the other side, there is still much space for investigation and improvement in our sharpening spatial attention module. Through visualization results, we believe that it is a meaningful point to introduce sharpening filters in spatial attention architectures. There definitely exist other suitable filter plans in spatial attention mechanism to play a certain role in some special applications of object detection.

## Figures and Tables

**Table 1:** Comparison of two different fusion methods of attention mechanisms.

Description	AP50	AP50:95
YOLOv5s	59.9%	35.5%
YOLOv5s+SE _(left)_	59.3%	34.9%
YOLOv5s+SE _(right)_	60.5%	35.9%
YOLOv5s+ECA _(left)_	59.4%	35.1%
YOLOv5s+ECA _(right)_	61.1%	36.0%
YOLOv5s+CBAM _(left)_	58.9%	34.5%
YOLOv5s+CBAM _(right)_	59.2%	34.8%

**Table 2:** Comparison of different combination methods of our SSAM (Confidence threshold: 0.001; NMS threshold: 0.6).

Description	Backbone	Neck	Head	AP50	AP50:95
YOLOv5s	No	No	No	59.9%	35.5%
YOLOv5s+ECA	No	No	ECA	60.7%	35.9%
YOLOv5s+SSA	No	No	SSA	60.4%	35.7%
YOLOv5s+SSAM	No	No	ECA+SSA	60.7%	35.7%
YOLOv5s+SE	SE	SE	SE	60.5%	35.9%
YOLOv5s+CBAM	CBAM	CBAM	CBAM	59.2%	34.8%
YOLOv5s+ECA	ECA	ECA	ECA	61.1%	36.0%
YOLOv5s+[ECA+SAM]	ECA	ECA	ECA+SAM	59.5%	35.1%
YOLOv5s+SSAM	SSA+ECA	SSA+ECA	SSA	False	False
YOLOv5s+SSAM	SSA+ECA	SSA+ECA	SSA+ECA	False	False
YOLOv5s+SSAM	ECA	SSA+ECA	SSA+ECA	61.4%	36.1%
YOLOv5s+SSAM	ECA	ECA	SSA+ECA	62.2%	36.8%
YOLOv5s+SSAM	ECA	ECA	ECA+SSA	62.3%	37.1%
YOLOv5s+SSAM	ECA	ECA	ECA+NSA	59.6%	35.0%
YOLOv5s+SSAM	ECA	ECA	SSA	60.6%	35.9%

**Table 3:** Comparison of different operators of edge detection in our SSA module.

Description	Laplace 3 × 3	Laplace 5 × 5	Sobel 3 × 3	AP50	AP50:95
YOLOv5s				59.9%	35.5%
YOLOv5s+SSAM	√			61.4%	36.4%
YOLOv5s+SSAM		√		62.3%	37.1%
YOLOv5s+SSAM			√	61.8%	36.5%

**Table 4:** Comparison of different extraction methods of our SSA module.

Description	Maxpool	Avgpool	Max and Avgpool	AP50	AP50:95
YOLOv5s				59.9%	35.5%
YOLOv5s+SSAM	√			61.3%	36.3%
YOLOv5s+SSAM		√		60.9%	36.0%
YOLOv5s+SSAM			√	62.3%	37.1%

**Table 5:** Comparison of different structures of our SSAM in YOLOv5s model.

Description	AP50	AP75	AP50:95	FPS	Gflops	Parameters	Weights
YOLOv5s	55.6%	39.0%	36.8%	455	17.0	7,276,605	14.11 m
YOLOv5s+SE	56.0%	40.1%	36.8%	416	17.1	7,371,325	14.30 m
YOLOv5s+SE+SSA	56.9%	39.8%	36.9%	416	17.1	7,371,406	14.31 m
YOLOv5s+ECA	56.7%	40.2%	37.0%	435	17.1	7,276,629	14.12 m
YOLOv5s+ECA+SSA	57.6%	40.9%	37.7%	435	17.1	7,276,710	14.13 m

**Table 6:** Comparison of different structures of our SSAM in YOLOv3-tiny model.

Description	AP50	AP75	AP50:95	FPS	Gflops	Parameters	Weights
YOLOv3-tiny	34.9%	15.8%	17.6%	667	13.3	8,852,366	16.94 m
YOLOv3-tiny+SE	35.7%	16.4%	18.1%	588	13.4	8,969,742	17.18 m
YOLOv3-tiny+SE+SSA	36.0%	16.8%	18.3%	588	13.4	8,969,796	17.19 m
YOLOv3-tiny+ECA	35.6%	16.4%	18.0%	625	13.3	8,885,155	17.01 m
YOLOv3-tiny+ECA+SSA	35.8%	16.5%	18.2%	625	13.3	8,885,209	17.02 m

**Table 7:** Comparison of different object size with our SSAM in YOLOv5s model.

Description	AP_small_	AP_medium_	AP_large_
YOLOv5s	21.1%	41.9%	45.5%
YOLOv5s+SE	20.9%	42.1%	46.5%
YOLOv5s+SE+SSA	21.7%	42.0%	46.6%
YOLOv5s+ECA	20.5%	42.4%	47.1%
YOLOv5s+ECA+SSA	23.1%	43.2%	47.8%

**Table 8:** Comparison of different object size with our SSAM in YOLOv3-tiny model.

Description	AP_small_	AP_medium_	AP_large_
YOLOv3-tiny	9.6%	22.2%	22.1%
YOLOv3-tiny+SE	9.8%	22.6%	22.8%
YOLOv3-tiny+SE+SSA	10.4%	23.0%	22.6%
YOLOv3-tiny+ECA	9.8%	22.6%	22.9%
YOLOv3-tiny+ECA+SSA	10.1%	22.5%	23.1%

## Data Availability

The datasets used in the paper VOC2012 and COCO2017 are all publicly available datasets. These data can be found here: [http://host.robots.ox.ac.uk/pascal/VOC/]; [http://cocodataset.org].
